# Hyperspectral volumetric coherent anti‐Stokes Raman scattering microscopy: quantitative volume determination and NaCl as non‐resonant standard

**DOI:** 10.1002/jrs.4876

**Published:** 2016-02-10

**Authors:** Arnica Karuna, Francesco Masia, Paola Borri, Wolfgang Langbein

**Affiliations:** ^1^ School of Physics and AstronomyCardiff University, The ParadeCardiffCF24 3AAUK; ^2^ School of BiosciencesCardiff UniversityMuseum AvenueCardiffCF10 3AXUK

**Keywords:** CARS microscopy, hyperspectral image analysis

## Abstract

In this work, we demonstrate quantitative volume determination of chemical components in three dimensions using hyperspectral coherent anti‐Stokes Raman scattering microscopy, phase‐corrected Kramers–Kronig retrieval of the coherent anti‐Stokes Raman scattering susceptibility and factorization into concentration of chemical components. We investigate the influence of the refractive index contrast between water and polymer beads (polystyrene and polymethylmethacrylate), showing that it leads mainly to concentration errors, while the spectral error is less affected. The volume of polystyrene beads of sizes from 200 nm to 3 *μ*m is determined with 10% relative error and 1% absolute error in the region of interest. We furthermore establish the use of sodium chloride as non‐resonant reference material free of Raman‐active vibrational resonances. © 2016 The Authors Journal of Raman Spectroscopy Published by John Wiley & Sons Ltd.

## Introduction

Raman micro‐spectroscopy is a fundamental tool in material science and cell biology because of the ability to map the distribution of the endogenous molecules in cells by measuring their vibrational spectra, hence without the need of introducing external molecules as labels. The applicability of spontaneous Raman scattering to cell imaging is limited by the small cross‐section of the process, which requires long integration times to reach a sufficient signal‐to‐noise ratio. Moreover, the scattered Raman field is normally measured in the Stokes region (at smaller energy than the excitation), which overlaps with the fluorescence emission of endogenous molecules. With the advent of pulsed lasers, high peak coherent light intensity could be achieved with tolerable average power, enabling the development of multiphoton microscopy. In the last decade coherent Raman scattering (CRS) techniques, for example, coherent anti‐Stokes Raman scattering (CARS), have been proposed as an alternative to spontaneous Raman.^1^ In CRS, two beams of light, the pump and Stokes with frequencies *ω*_p_ and *ω*_S_, respectively, drive the material at their difference frequency, *ω*_p_−*ω*_S_. In typical two‐pulse CARS, the vibrational response is probed by the pump resulting in a coherent emission at2*ω*_p_−*ω*_S_, blue shifted from the excitation, allowing spectral discrimination from the background endogenous fluorescence. Differently from spontaneous Raman scattering, in CRS, all the molecules in the focal volume are driven coherently, and the emitted CARS intensity scales with the square of the number of identical molecules. The chemical specificity and sensitivity as well as inherent sectioning capability of hyperspectral CRS microscopy reinforce its usability as a label‐free imaging tool for biology and medical sciences. 2, 3, 4, 5

While the CARS intensity could naively be expected to have a maximum when *ω*_p_−*ω*_S_ matches a vibrational frequency of the molecule under investigation, the coherent nature of the emission and the presence of non‐resonant responses leads to an interference, which is modifying the lineshape. The CARS intensity can be described as *I*_C_∝|*χ*|^2^=|*χ*_R_+*χ*_NR_|^2^, where *χ*_R_(*χ*_NR_) is the resonant (non‐resonant) component of third‐order susceptibility corresponding to the CARS process. *χ*_R_ has both real and imaginary components, while *χ*_NR_ is due to a purely electronic contribution and is real for materials without two‐photon absorption, that is, having a band‐gap much larger than the two‐photon energy. This is the case for water, proteins and DNA for excitation pulses in the biological window. Two‐photon absorption would also lead to photodamage, limiting the applicability of CRS. Therefore, *I*_C_∝|*χ*_R_|^2^+2*R*(*χ*_R_ *χ*_NR_)+|*χ*_NR_|^2^.^6^ The imaginary component of the resonant CARS susceptibility, that is, *I*(*χ*) = *I*(*χ*_R_) resembles the spontaneous Raman scattering cross‐section and is linear in the concentration of the detected chemical species. The challenge in analyzing the CARS intensity data is the determination of the phase of *χ*. However, the presence of the non‐resonant background is also an advantage, as it enhances the intensity of weak *χ*_R_ by the homodyning in the interference term 2*R*(*χ*_R_ *χ*_NR_), and it allows to use a non‐resonant material as reference. For the phase retrieval, effectively equivalent techniques^7^ in current use are based on a time domain Kramers–Kronig (KK) formulation^8^

and the maximum entropy method.^9^ Recently, we have developed a phase‐corrected KK approach (PCKK), which is improving on the KK formulation, by correcting not only the phase but also the amplitude of the signal, and results in a quantitative susceptibility in units of *χ*_ref_ of a known non‐resonant reference material, $\bar{\chi }=\chi /\chi_{\text{ref}}$.^10^ Typically, glass is used as reference material as it is part of the microscope sample. Recently, Camp *et al*.^11^ discussed the variability in the results of the KK method using different glasses as reference material, because of the presence of resonant contributions in the glass vibrational spectrum in the 500–750 cm^−1^ region, which depends on the specific formulation of the glass. We discuss in the last section the use of salt (NaCl) as alternative reference material without these issues. Once the complex susceptibility is obtained, the resulting hyperspectral images still need to be analyzed in terms of the physical quantities of interest, which is the chemical composition of the specimen as a spatially resolved map of few spectral components. Different techniques have been proposed in literature based on principal component analysis,^12^ hierarchical cluster analysis,^13^ independent component analysis,^14^ classical least squares analysis,^15^ multivariate curve resolution analysis^16^ and phasor analysis.^17^ We have recently developed an algorithm to analyze CARS hyperspectral images, which determines the quantitative spectra and the absolute concentration images of chemical components without prior knowledge of the spectra, which we call factorization into spectra and concentrations of chemical components (FSC^3^). 10, 18 In this method, the hyperspectral data are factorized as $D=\underset{i}{\sum }C_{i}\times S_{i}+E$, with **C**_*i*_ and **S**_*i*_ being the non‐negative concentration distribution and spectrum of the *i*‐th component, and **E** is the error, whose norm is minimized.

In this paper, we demonstrate the applicability of our hyperspectral CARS image analysis method for quantitative determination of the absolute volumes of chemical components in three‐dimensional imaging. As a proof of principle, we have investigated polystyrene (PS) and polymethylmethacrylate (PMMA) beads of known size. We discuss the error in the factorization algorithm observed at the interface of the beads due to light refraction caused by the change in the refractive index between the bead and the surrounding. Using the FSC^3^ method, we have evaluated the volume of the beads and found a good agreement with the nominal values, also for bead of size below the system resolution. Additionally, as already mentioned, we suggest sodium chloride (NaCl) as a non‐resonant reference material alternative to commonly used glass.

## Experiment

Hyperspectral CARS images have been acquired in our home‐built multi‐modal microscope set‐up^19^. Briefly, we split the broadband beam (660–970 nm) from a 5fs Ti:Sapphire laser (Venteon Pulse One PE) with 80MHz repetition rate into the pump beam (660–730 nm) and the Stokes beam (730–900 nm). We use spectral focussing 20, 21, 22, 23 in which an equal linear chirp is applied to both the pump and Stokes beams, resulting in a constant instantaneous frequency difference (IFD), which can be tuned by varying the delay between pump and Stokes beams. The resulting pump pulse duration at the sample is about 1.5ps, resulting in a IFD resolution of about 20cm^−1^. The CARS signal is collected by the condenser, separated from the excitation beams using two Semrock FF01‐562/40 filters and detected with a photomultiplier (Hamamatsu H7422‐40). The data presented in this paper were acquired using either a 20× 0.75NA air objective and a 0.72NA air condenser, or a 60× 1.27NA water immersion objective (Nikon CFI Plan Apochromat IR *λ*S series) with a 1.4NA oil immersion condenser. The pixel dwell times were 10*μ*s unless otherwise specified, and typical powers at the sample were a few mW for each beam.

Three‐dimensional CARS images of PS and PMMA beads of various sizes have been acquired as a function of the IFD in the CH stretch region (2400–3600) cm^−1^ with a IFD step size of 3cm^−1^. PS and PMMA beads were dispersed in a 2*%* agar solution, which was pipetted into a well, created by the 9 mm diameter opening of a 0.12 mm thick adhesive imaging spacer (Grace BioLabs) attached to a microscope slide, and subsequently capped with a #1 coverslip. Sodium chloride crystals were measured across the maximum IFD range of our set‐up, (1200–3700) cm^−1^. Sodium chloride crystals were obtained starting from a super‐saturated NaCl–water solution, which was prepared by dissolving high purity (>99.5*%*) sodium chloride (Sigma Aldrich) in distilled water. The solution was drop cast on a microscope slide. A #1 coverslip was placed on the solution, to provide an optically flat structure, and the water was allowed to slowly evaporate at 4°C for several days. The IFD step size for those measurements was set to 5cm^−1^.

## Results and Discussion

### Quantitative determination of the volume of PS and PMMA beads

**Figure 1:**
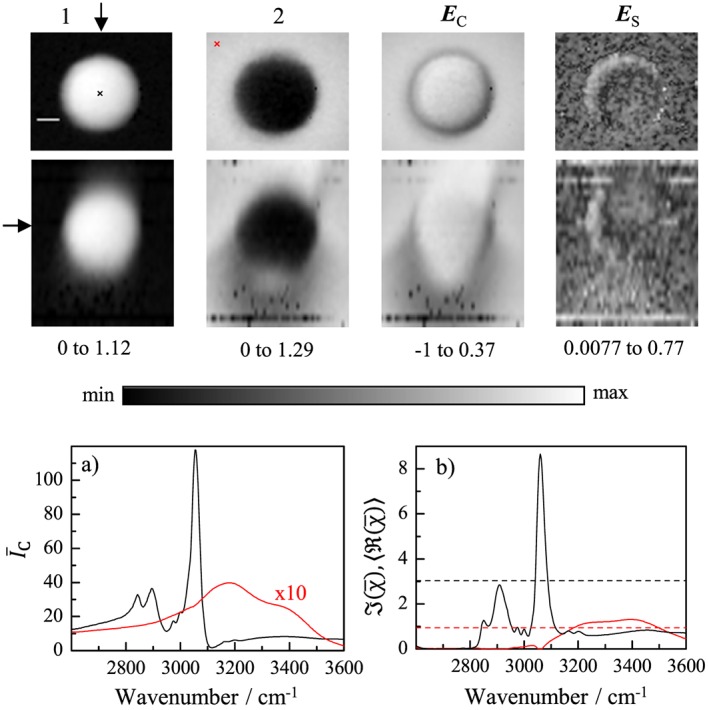
(Colour online) Results of a FSC^3^ analysis of CARS hyperspectral volumetric images of a 3*μ*m bead taken with about 10 (6)mW pump (Stokes) power at the sample. Top: *xy* distribution of the concentrations of the FSC^3^ components 1 and 2, and the concentration error **E**_C_ on a linear grayscale and the spectral error **E**_S_ on a logarithmic grayscale. Middle: Corresponding *xz* sections. The *z* and *y*‐positions of the *xy* and *xz* sections are indicated by an arrow in the *xz* and *xy* sections. The grayscales ranges are given below the images. The scale bar is 1*μ*m. a) CARS ratio ${Ī}_{C}$ spectra of water (red) and the PS bead (black) taken at positions indicated by the cross in the top left images. The maximum detected CARS intensity *I*_C_ in polystyrene was about 10^8^ photoelectrons/s. b) FSC^3^ spectra of component 1 (black) and component 2 (red), with $J(\bar{\chi })$ and $ℜ(\bar{\chi })$ as solid and dashed lines, respectively.

**Figure 2:**
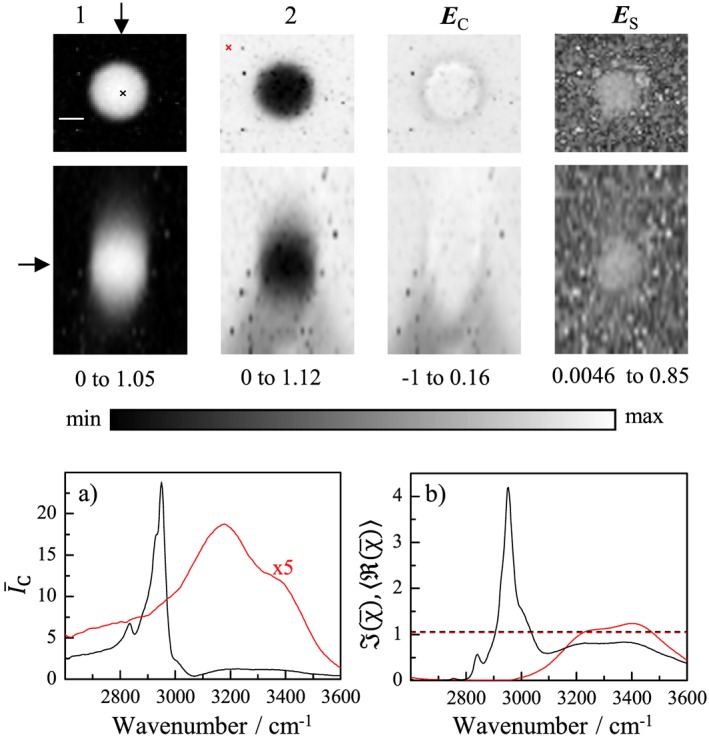
(Colour online) Same as Fig.1 for a 2.6*μ*m PMMA bead. The maximum CARS intensity in PMMA was about 2 × 10^7^ photoelectrons/s.

The hyperspectral images of the PS and PMMA beads have been analyzed following the procedure described by Masia *et al*.^[^ 10 ^]^ First, the data have been denoised by singular value decomposition. To correct the measured spectrum by the transduction coefficient of the set‐up, the CARS intensity *I*_C_ is normalized using the intensity *I*_ref_ measured in a non‐resonant material, in our case, the coverslip glass, yielding ${Ī}_{C}=I_{C}/I_{\text{ref}}$. We move a few tens of *μ*m into the glass to ensure negligible overlap of the point‐spread function (PSF) with other materials while limiting the distortion of the beam focus by the change in spherical errors, which would lead to a reduced CARS signal. The CARS ratio spectra given in Figs  1(a) and  2(a) show the typical asymmetric lineshape of resonances due to the interference with the non‐resonant background. We then retrieve the calibrated complex susceptibility $\bar{\chi }$ using PCKK on ${Ī}_{C}$. The resulting hyperspectral $J(\bar{\chi })$ and the spectrally averaged $ℜ(\bar{\chi })$ data are then decomposed into two chemical components and their spectra using FSC^3^.

In Fig. 1, we show the results of the FSC^3^ analysis on the CARS hyperspectral data of a PS bead with nominal size of 3*μ*m acquired using the 1.27NA objective. The two components obtained are identified as water and PS by their spatial distribution and $J(\bar{\chi })$ spectra. In the FSC^3^ method, we find the volume concentrations and spectral normalization of the different components imposing that the deviation of sum concentration from unity, that is, the concentration error $\left||E_{C}||=||\underset{i}{\sum }C_{i}-1|\right|$, is minimum. The spatial distribution of **E**_C_ is shown in Fig. 1, with the highest |*E*_C_|(apart from random pixels due to laser fluctuations, which have been excluded from the analysis) found at the edge of the bead and in the wake of the bead (the excitation beams enter from the top in this image). The tilt of the wake indicates a slight misalignment of the excitation beams in the objective back focal plane. We attribute the concentration error to the distortion of the excitation beams by the refraction at the water to polymer interface, with a refractive index step from 1.33 to 1.59. The effects of the refractive index contrast of beads on the CARS intensity and spectra have been investigated before. 24, 25 Here, we determine its influence on the retrieved spectra of the susceptibility after PCKK and the FSC^3^ factorization. The concentration error is −24% at the bead edge and +10% in the centre of the bead, considering the *xy* plane in the middle of the bead. The spectral error **E**_S_ representing the error in the factorization^10^ is also shown. It is also maximum at the edge of the bead (∼10*%*) but generally remains small in the few % range. The full 3D data are available at http://dx.doi.org/10.17035/d.2015.0008102789. In biological samples, we have seen that the spectral error can vary from ∼30*%* at large droplets of adipocytes 10, 26 to less than 1*%* when cell without large lipid droplets are imaged.^18^ The negative concentration error at the edges and in the wake of the bead comes from the distortion of the excitation beams, reducing the intensity in the focus and thus the CARS signal. The positive concentration error in the middle of the bead comes from the better focussing in the higher index PS bead, which acts as solid immersion lens. We can also see the positive concentration error in the water in front of the bead, which we attribute to the reflected beams by the water bead interface. Note that the interface has a reflection amplitude of 9%. Qualitatively similar effects can be seen when measuring lipid droplets in cells. 10, 26

We then investigated PMMA beads, which have a refractive index of 1.49, similar to typical lipids (1.43–1.49), and thus less refractive index contrast to water than PS. The results on a 2.6*μ*m PMMA bead are given in Fig. 2 and shows two components with FSC^3^ spectra corresponding to water and PMMA. We can note a significant fraction of water also in the PMMA related component. This is due to the smaller size of the bead. Note that the PSF related to the concentration is a coherent field PSF, not the CARS intensity PSF typically quoted as resolution in the literature. The field PSF has a larger width than the intensity PSF. For the 0.2*μ*m PS bead shown later, we found a full‐width‐at‐half‐maximum (FWHM) of the concentration distribution of 0.32*μ*m laterally and 0.93*μ*m axially. Therefore, a relevant part of the field PSF is in water even when imaging the centre of the bead. The concentration error due to refraction is, as expected, much smaller as compared to PS, about −3% at the edge of the bead and +6% at the centre, for the *xy* plane through the middle of the bead.

**Figure 3:**
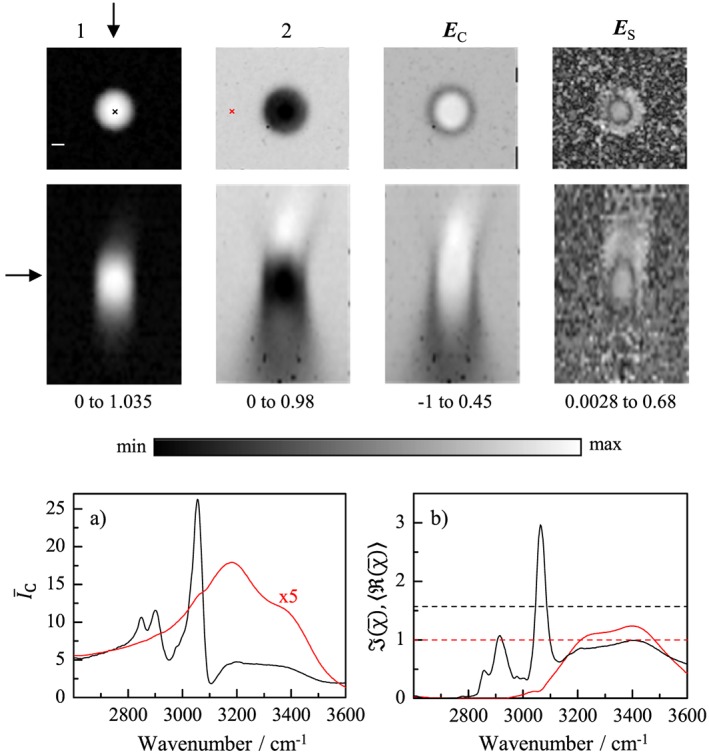
(Colour online) Same as Fig.1 for measurements obtained using a 0.75NA objective, fill factor 0.55, and a 0.72NA condenser, and 15 (10)mW pump (Stokes) powers at the sample. The maximum detected CARS intensity in the PS bead was about 5 × 10^7^ photoelectrons/s.

We now investigate the effect of the numerical aperture of the imaging. Instead of 1.27NA in excitation, fill factor 0.98 and 1.4NA in collection, which is corresponding to an opening angle of 73° excitation and 90° detection, we use 0.75NA in excitation, fill factor 0.55, and 0.72NA in collection, corresponding to an opening angle of 18° in excitation and 33° in detection. In Fig. 3, we show the results of the FSC^3^ analysis of a hyperspectral CARS image of a 3*μ*m PS bead. The observed concentration error at the edge of the bead is −25%, similar to the 1.27 NA case (Fig. 1), but it is larger, +29%, at the centre of the bead. The smaller excitation NA results in a larger field PSF extension, resulting in more water in the PS spectra. Furthermore, the refraction effects are more relevant because the opening angle is smaller compared with the refraction angles.

**Figure 4:**
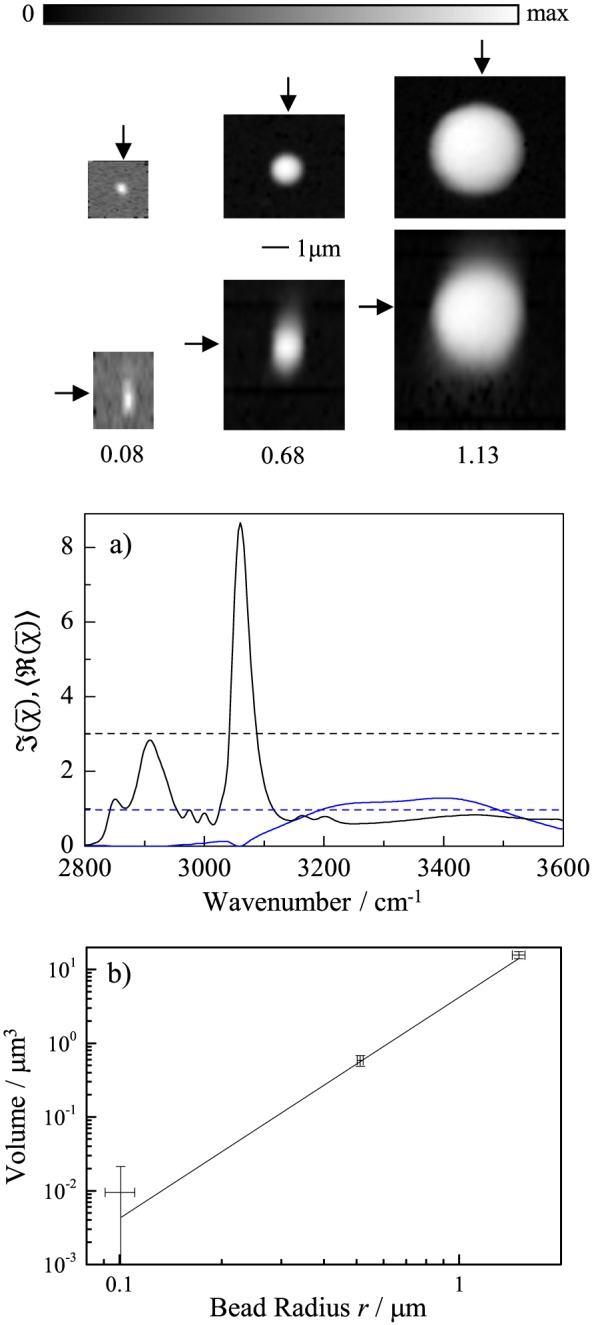
(Colour online) Results of the FSC^3^ analysis of coherent anti‐Stokes Raman scattering hyperspectral images of PS beads of various nominal sizes. The top (middle) row shows *xy* (*xz*) maps of the concentration of the PS component, at the *z* (*y*) positions indicated by the arrows. a) FSC^3^ spectra of polystyrene (black) and water (blue) components b) volumes of beads measured from the FSC^3^ concentrations (*V*_PS_, symbols, error bars see text) and their nominal sizes (solid line), versus nominal bead radius.

To verify the accuracy of the volume determination, we performed CARS measurements on PS beads of nominal sizes (3.004 ± 0.065*μ*m), (1.02 ± 0.01*μ*m) and (0.20 ± 0.01*μ*m). CARS data for 0.2*μ*m beads were acquired using a pixel dwell time of 100*μ*s. The hyperspectral data have been analyzed together using FSC^3^ to obtain a common spectral basis. The results are shown in Fig. 4.

The volumes of the beads can be calculated using the concentration maps corresponding to PS obtained by the FSC^3^ analysis. First, we create a bounding cuboid of volume *V*, with the lateral edges located at ±(*r* + *δ* *r*) from the centre of the bead, where *r* is the nominal radius of the bead and *δ* *r* = 0.28*μ*m is an additional width to accommodate the PSF size. The axial limits of the cuboid are given by ±(*r* + *δ* *z*), where *δ* *z* = 0.9*μ*m. To determine the absolute PS volume, we furthermore determine the volume fraction *γ* = 0.77 of PS in the PS component using the ratio between the $J(\bar{\chi })$ spectrum of the component to that of bulk PS.^10^ We evaluate the background concentration *C*_w_ of a few percent as average of the PS component in spatial regions corresponding to water and find the total volume of the PS bead as $V_{\text{PS}}=\gamma \underset{V}{\int }(C_{\text{PS}}-C_{w})\mathrm{dV}$. We estimate the error of *V*_PS_ by $\sqrt{{\left(0.1\times V_{\text{PS}}\right)}^{2}\;+\;{\left(0.01\times V\right)}^{2}}$, considering a 10% relative error and a 1% absolute error of the concentration. The calculated volumes are shown in Fig. 4b as a function of the nominal size of the beads. The obtained values are consistent with the volumes calculated considering the nominal sizes, even for the smallest bead (*r* = 0.1*μ*m), which is smaller than the resolution. The results demonstrate the capability of hyperspectral CARS imaging and FSC^3^ analysis to quantitatively determine the chemical composition of structures in three dimensions.

**Figure 5:**
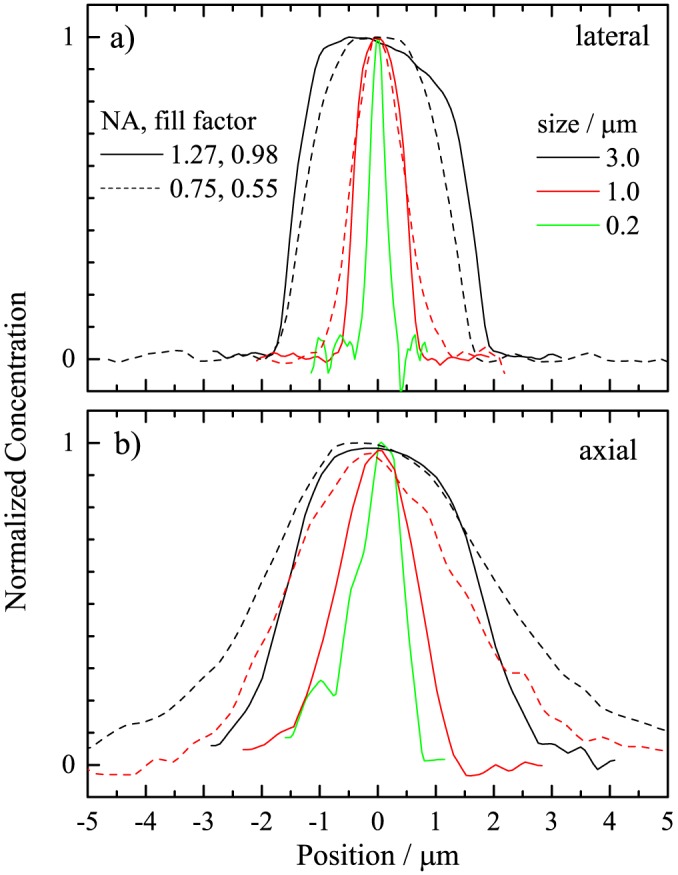
(Colour online) Normalized concentration profiles of PS beads in lateral (a) and axial (b) direction. Optics: 1.27NA (solid lines, fill factor 0.98), 0.75NA (dashed lines, fill factor 0.55). Bead sizes: 3*μ*m (black), 1*μ*m (red) and 0.2*μ*m (green).

In Fig. 5, we show the concentration profiles of PS beads using the two objective/condenser combinations specified earlier. For the 1.27NA optics, we find for the 0.2*μ*m bead data a lateral FWHM of 0.31*μ*m and an axial FWHM of 0.9*μ*m. For the 0.75NA optics, we find for the 1*μ*m bead a lateral FWHM of 1.0*μ*m and an axial FWHM of 3.3*μ*m. Note that the axial scale should be multiplied with the refractive index of water for this dry objective, resulting in a FWHM of 4.4*μ*m. It is expected that the lateral resolution scales as the NA, while the axial resolution scales as NA^2^. The different fill factors complicate this scaling – using an effective NA ratio of 2.5 the observed behaviour is consistent with the expectation.

### Sodium chloride as non‐resonant reference material

The data in the previous section have been analyzed using cover slip glass (Schott D263M, about 65% fused silica, plus a set of other oxides) as non‐resonant reference material. This material has vibrations at frequencies below 1000cm^−1^, which are not relevant for our set‐up, which is interrogating only frequencies above 1200cm^−1^. However, using glass at lower IFDs does introduce artefacts as reported by Camp *et al*.^11^. Moreover, coverslip glass is an amorphous material with a composition varying between different manufacturers. Different glass types, even those having comparable refractive index, have therefore different vibrational spectra, hindering their applicability as universal non‐resonant material for CARS data analysis.

Here, we propose the use of NaCl as standard reference non‐resonant material. NaCl has Raman‐inactive longitudinal optical (LO) and transverse optical (TO) phonon modes^27^ at ∼170 cm^−1^ and ∼270 cm^−1^. Second‐order Raman scattering has been observed for wavenumbers <400 cm^−1^ but is expected to be negligible in CARS. Furthermore, the bandgap of NaCl is 9eV,^28^ much larger than the excitation photon energy of 1‐1.7eV in the "biological window" typically used in CARS. The background due to electronic four‐wave mixing is therefore far off resonance, and we expect the third‐order susceptibility to be effectively independent of the photon energies in the biological window.

**Figure 6:**
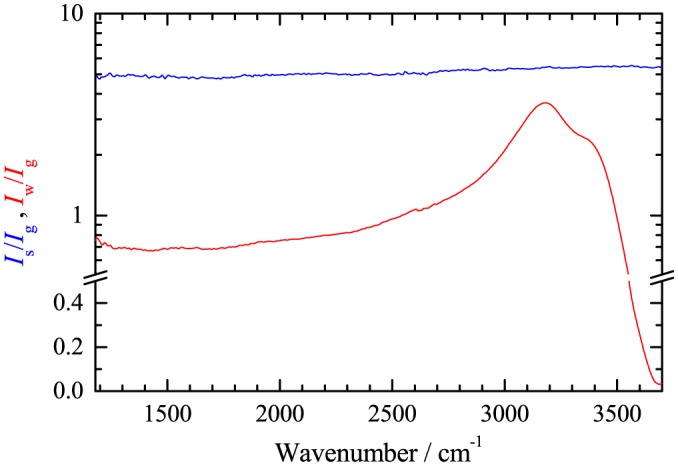
(Colour online) CARS intensity ratio spectra between NaCl and glass *I*_s_/*I*_g_ (blue) and between water and glass *I*_w_/*I*_g_ (red).

In Fig. 6, we show the ratio of the CARS intensities measured in a NaCl crystal (*I*_s_) and a glass slide (*I*_g_). The data have been acquired using the 0.75NA objective and the 0.72NA condenser. The ratio shows a weak frequency dependence in the range 1200–3700cm^−1^ with about 10% variation. This variation is similar to the estimated measurement systematics. The measured value can be compared with the calculated ratio ${\left(\chi_{s}^{\left(3\right)}/\chi_{g}^{\left(3\right)}\right)}^{2}$, assuming that the nonlinearity is given by the Kerr effect. The third‐order susceptibility can be expressed as *χ* ^(3)^∝*n*_0_ *n*_2_, where *n*_0_ is the linear refractive index and *n*_2_ is the nonlinear refractive index.^29^ Considering the *n*_2_ values 1.59 × 10^−13^ esu of NaCl and 0.85 × 10^−13^ esu for fused silica measured using non‐degenerate four‐wave mixing experiments with 3 nanosecond pulses of ∼1*μ*m wavelength at 60cm^−1^ difference,^29^ and the *n*_0_ values 1.539 for NaCl^30^ and 1.456 for fused silica^31^ at the central wavelength of the pump beam, which we consider as average wavelength of the fields involved in the non‐linearity, we expect a CARS intensity ratio of 3.9, in reasonable agreement with the measured value of about 5. We can expect that the $\chi_{s}^{\left(3\right)}$ does not change for IFDs down to the Brillouin scattering range below 10cm^−1^.

Using NaCl as reference material has the disadvantage that it is not present in a typical sample studied. However, water (or buffer) and glass is typically present in biological samples. A calibration procedure should therefore contain a measurement of the NaCl to glass ratio for the specific glass type used, as well as a glass to buffer ratio. In this way, one can use the glass or the buffer measured within the data as reference, by correcting it to the NaCl reference. The ratio between the measured CARS intensity in water (*I*_w_) and the CARS intensity measured in glass is shown in Fig. 6. It shows the OH resonance around 3200cm^−1^ and reduces close to zero at 3700cm^−1^ due to the out of phase contributions between resonant and non‐resonant parts.

## Conclusions

In this work, we have shown that volumetric hyperspectral CARS microscopy combined with PCKK phase retrieval and FSC^3^ factorization determines quantitative volume concentrations and spectra. The accuracy is limited on the one hand by the signal‐to‐noise ratio, and on the other hand by the influence of the refractive index structure in the sample. The latter leads mostly to systematic relative error in the concentration due to the influence on the beam focus, and thus the CARS intensity. The retrieved spectra are less affected as the refraction is only weakly dependent on the excitation beam wavelength. Typical resulting concentration errors for samples as the ones presented here are 1% absolute mostly due to the limited signal to noise, and 10% relative due to the influence of refractive index structure.

We have furthermore proposed sodium chloride as a suitable non‐resonant CARS reference material free of vibrational resonances above 10cm^−1^ and measured its CARS ratio to glass in the range 1200‐3700cm^−1^. With a band‐gap of 9eV, it is also free of electronic resonances for excitation pulses in the biological window. Considering that it is widely available and forms cubic crystals with uniform composition using a simple preparation method, NaCl represent a convenient standard medium for quantitative calibration of measured CARS responses. The data presented in this work are available from the Cardiff University data archive under http://dx.doi.org/10.17035/d.2015.0008102789.
